# Supplementary material for: Risk Factors for Anticancer Drug-Induced Hyponatremia: An Analysis Using the Japanese Adverse Drug Report (JADER) Database
Source: Medicina (Kaunas). 2023 Jan 13;59(1):166. doi: 10.3390/medicina59010166 (PMC9860742; doi:10.3390/medicina59010166)
Supplement: Supplementary file 1 [file medicina-59-00166-s001.zip › medicina-2066067-supplementary.pdf]

Adverse events	PT codes
hyponatremia	10002776 Antidiuretic hormone abnormality, 10005332 Blood antidiuretic hormone abnormal, 10005335 Blood antidiuretic hormone increased, 10005800 Blood sodium abnormal, 10005802 Blood sodium decreased, 10014149 Ectopic antidiuretic hormone secretion, 10021036 Hyponatremia, 10075865 Hyponatremic coma, 10066151 Hyponatremic encephalopathy, 10021037 Hyponatremic syndrome, 10074867 Hypoosmolar state, 10053198 Inappropriate antidiuretic hormone secretion, 10069350 Osmotic demyelination syndrome, 10070604 Rapid correction of hyponatremia

Esophageal cancer	10030176 Esophageal neoplasms malignant, 10030137 Esophageal adenocarcinoma, 10030140 Esophageal adenocarcinoma recurrent, 10030141 Esophageal adenocarcinoma stage 0, 10030142 Esophageal adenocarcinoma stage I, 10030143 Esophageal adenocarcinoma stage II, 10030144 Esophageal adenocarcinoma stage III, 10030145 Esophageal adenocarcinoma stage IV, 10030155 Esophageal carcinoma, 10030159 Esophageal carcinoma recurrent, 10030162 Esophageal carcinoma stage 0, 10030187 Esophageal squamous cell carcinoma recurrent, 10030188 Esophageal squamous cell carcinoma stage 0, 10030189 Esophageal squamous cell carcinoma stage I, 10030190 Esophageal squamous cell carcinoma stage II, 10030191 Esophageal squamous cell carcinoma stage III, 10030192 Esophageal squamous cell carcinoma stage IV, 10055102 Esophageal cancer metastatic, 10058527 Esophageal squamous cell carcinoma metastatic, 10061534 Esophageal squamous cell carcinoma, 10082968 Esophageal adenosquamous carcinoma, 10086585 Neuroendocrine carcinoma of the esophagus
Lung cancer	10001245 Adenosquamous cell lung cancer, 10001247 Adenosquamous cell lung cancer recurrent, 10001248 Adenosquamous cell lung cancer stage 0, 10001249 Adenosquamous cell lung cancer stage I, 10001250 Adenosquamous cell lung cancer stage II, 10001251 Adenosquamous cell lung cancer stage III, 10001254 Adenosquamous cell lung cancer stage IV, 10023774 Large cell lung cancer, 10023775 Large cell lung cancer recurrent, 10023776 Large cell lung cancer stage 0, 10023777 Large cell lung cancer stage I, 10023778 Large cell lung cancer stage II, 10023779 Large cell lung cancer stage III, 10023780 Large cell lung cancer stage IV, 10025031 Lung adenocarcinoma, 10025033 Lung adenocarcinoma recurrent, 10025034 Lung adenocarcinoma stage 0, 10025035 Lung adenocarcinoma stage I, 10025036 Lung adenocarcinoma stage II, 10025037 Lung adenocarcinoma stage III, 10025038 Lung adenocarcinoma stage IV, 10025120 Lung squamous cell carcinoma recurrent, 10025121 Lung squamous cell carcinoma stage 0, 10025122 Lung squamous cell carcinoma stage I, 10025123 Lung squamous cell carcinoma stage II, 10025124 Lung squamous cell carcinoma stage III, 10025125 Lung squamous cell carcinoma stage IV, 10029515 Non-small cell lung cancer

	recurrent, 10029516 Non-small cell lung cancer stage 0, 10029517 Non-small cell lung cancer stage I, 10029518 Non-small cell lung cancer stage II, 10029519 Non-small cell lung cancer stage III, 10029520 Non-small cell lung cancer stage III, 10029521 Non-small cell lung cancer stage III, 10029522 Non-small cell lung cancer stage IV, 10041826 Squamous cell carcinoma of lung, 10059515 Non-small cell lung cancer metastatic, 10061873 Non-small cell lung cancer, 10069730 Large cell lung cancer metastatic,10071533 Lung squamous cell carcinoma metastatic, 10080717 Primary pulmonary melanoma, 10081400 Sarcomatoid carcinoma of the lung, 10084787 HER2 mutant non-small cell lung cancer
Renal cancer	10038486 Renal neoplasms, 10009253 Clear cell sarcoma of the kidney, 10010428 Congenital cystic kidney disease, 10010607 Congenital renal cyst, 10027455 Metastases to kidney, 10029145 Nephroblastoma, 10033702 Papillary tumor of renal pelvis, 10038389 Renal cancer, 10038390 Renal cancer recurrent, 10038391 Renal cancer stage I, 10038392 Renal cancer stage II, 10038393 Renal cancer stage III, 10038394 Renal cancer stage IV, 10038410 Renal cell carcinoma recurrent, 10038411 Renal cell carcinoma stage I, 10038412 Renal cell carcinoma stage II, 10038413 Renal cell carcinoma stage III, 10038414 Renal cell carcinoma stage IV, 10039019 Rhabdoid tumor of the kidney, 10050018 Renal cancer metastatic, 10050176 Renal oncocytoma, 10050513 Metastatic renal cell carcinoma, 10051948 Renal adenoma, 10058596 Renal cyst infection, 10059846 Renal cyst hemorrhage, 10061001 Benign renal neoplasm, 10061482 Renal neoplasm, 10061872 Non-renal cell carcinoma of kidney, 10067946 Renal cell carcinoma, 10069359 Leukemic infiltration renal, 10069908 Renal hemangioma, 10073251 Clear cell renal cell carcinoma, 10078493 Papillary renal cell carcinoma, 10080544 Chromophobe renal cell carcinoma, 10084074 Senior-Loken syndrome, 10085663 Clear cell papillary renal cell carcinoma
Stomach cancer	10017812 Gastric neoplasms malignant, 10001150 Adenocarcinoma gastric, 10017758 Gastric cancer, 10017761 Gastric cancer recurrent, 10017762 Gastric cancer stage 0, 10017763 Gastric cancer stage I, 10017764 Gastric cancer stage II, 10017765 Gastric cancer stage III, 10024520 Linitis plastica, 10055008 Gastric sarcoma, 10061967 Gastric cancer stage IV, 10062878 Gastroesophageal cancer, 10063916 Metastatic gastric cancer,

	10066896 HER2 positive gastric cancer, 10081398 Gastroesophageal cancer recurrent
Prostate cancer	10036908 Prostatic neoplasms malignant, 10036909 Prostate cancer metastatic, 10036911 Prostate cancer recurrent, 10036912 Prostate cancer stage 0, 10036917 Prostate cancer stage I, 10036918 Prostate cancer stage II, 10036919 Prostate cancer stage III, 10036920 Prostate cancer stage IV, 10060862 Prostate cancer, 10062904 Hormone-refractory prostate cancer, 10071119 Hormone-dependent prostate cancer, 10082915 Neuroendocrine carcinoma of prostate
Large intestine cancer	10010023 Colorectal neoplasms malignant (HLT), 10001167 Adenocarcinoma of colon, 10009944 Colon cancer, 10009952 Colon cancer recurrent, 10009953 Colon cancer stage I, 10009954 Colon cancer stage II, 10009955 Colon cancer stage III, 10009956 Colon cancer stage IV, 10010030 Colorectal cancer recurrent, 10010032 Colorectal cancer stage I, 10010033 Colorectal cancer stage II, 10010034 Colorectal cancer stage III, 10010035 Colorectal cancer stage IV, 10010038 Colorectal carcinoma stage 0, 10038019 Rectal adenocarcinoma, 10038038 Rectal cancer, 10038046 Rectal cancer recurrent, 10038047 Rectal cancer stage 0, 10038048 Rectal cancer stage I, 10038049 Rectal cancer stage II, 10038050 Rectal cancer stage III, 10038051 Rectal cancer stage IV, 10038086 Rectosigmoid cancer, 10038094 Rectosigmoid cancer recurrent, 10038095 Rectosigmoid cancer stage 0, 10038096 Rectosigmoid cancer stage I, 10038097 Rectosigmoid cancer stage II, 10038098 Rectosigmoid cancer stage III, 10038099 Rectosigmoid cancer stage IV, 10052358 Colorectal cancer metastatic, 10052360 Colorectal adenocarcinoma, 10055097 Rectal cancer metastatic, 10055114 Colon cancer metastatic, 10061237 Malignant anorectal neoplasm, 10061451 Colorectal cancer, 10063523 Colon cancer stage 0, 10069728 Rectosigmoid cancer metastatic, 10073359 Adenocarcinoma of appendix, 10073360 Appendix cancer, 10073361 Mucinous adenocarcinoma of appendix, 10073362 Undifferentiated carcinoma of colon, 10078934 Recurrent N-ras mutation-positive colorectal carcinoma, 10085146 Appendix cancer metastatic, 10059140 Large intestine carcinoma

Breast cancer	10066206 Apocrine breast carcinoma, 10006187 Breast cancer, 10057654 Breast cancer female, 10006189 Breast cancer in situ,10061020 Breast cancer male, 10055113 Breast cancer metastatic, 10006198 Breast cancer recurrent, 10006199 Breast cancer stage I, 10006200 Breast cancer stage II, 10006201 Breast cancer stage III, 10006202 Breast cancer stage IV, 10068582 Breast sarcoma, 10068583 Breast sarcoma metastatic, 10068584 Breast sarcoma recurrent, 10014437 Electron radiation therapy to breast, 10015721 Extended radical mastectomy, 10017677 Gamma radiation therapy to breast, 10083232 HER2 negative breast cancer, 10065430 HER2 positive breast cancer, 10085561 Hormone receptor negative HER2 positive breast cancer, 10085481 Hormone receptor positive HER2 negative breast cancer, 10083234 Hormone receptor positive breast cancer, 10076935 Hormone refractory breast cancer, 10021977 Inflammatory carcinoma of breast recurrent, 10021978 Inflammatory carcinoma of breast stage III, 10021979 Inflammatory carcinoma of breast stage IV, 10021980 Inflammatory carcinoma of the breast, 10073540 Intraductal papillary breast neoplasm, 10073094 Intraductal proliferative breast lesion, 10075713 Invasive breast carcinoma, 10073095 Invasive ductal breast carcinoma, 10073096 Invasive lobular breast carcinoma, 10073098 Invasive papillary breast carcinoma, 10073099 Lobular breast carcinoma in situ, 10062051 Malignant nipple neoplasm, 10053129 Malignant nipple neoplasm female, 10053128 Malignant nipple neoplasm male, 10026878 Mastectomy, 10027095 Medullary carcinoma of breast, 10073100 Metaplastic breast carcinoma, 10027799 Modified radical mastectomy, 10073101 Mucinous breast carcinoma, 10073103 Neuroendocrine breast tumor, 10054054 Estrogen receptor assay positive, 10033364 Paget's disease of nipple, 10034949 Photon radiation therapy to breast, 10036390 Postmastectomy lymphoedema syndrome, 10081036 Primary breast lymphoma, 10054057 Progesterone receptor assay positive, 10037773 Radical mastectomy, 10062090 Radiotherapy to breast, 10040700 Simple mastectomy, 10079307 Squamous cell breast carcinoma, 10075566 Triple negative breast cancer, 10083233 Triple positive breast cancer, 10073104 Tubular breast carcinoma, 10048199 X-ray therapy to breast
